# (*E*)-2-[(2-Ethyl­phen­yl)iminiometh­yl]-6-hydroxy­phenolate

**DOI:** 10.1107/S1600536810002503

**Published:** 2010-01-27

**Authors:** Serap Yazıcı, Çiğdem Albayrak, İsmail Gümrükçüoğlu, İsmet Şenel, Orhan Büyükgüngör

**Affiliations:** a Department of Physics, Faculty of Arts and Sciences, Ondokuz Mayıs University, TR-55139 Kurupelit-Samsun, Turkey; b Sinop Faculty of Education, Sinop University, TR-57000 Sinop, Turkey; c Department of Chemistry, Ondokuz Mayıs University, TR-55139 Kurupelit-Samsun, Turkey

## Abstract

The mol­ecule of the title compound, C_15_H_15_NO_2_, crystallizes in a zwitterionic form, and displays an *E* configuration about the C=N bond. The dihedral angle between the two aromatic rings is 5.59 (6)°. An intra­molecular N—H⋯O hydrogen bond generates an *S*(6) ring motif. In the crystal structure, pairs of mol­ecules are linked into centrosymmetric *R*_2_^2^(10) dimers by pairs of O—H⋯O hydrogen bonds. Aromatic π–π inter­actions are observed between the benzene rings of adjacent dimers [centroid–centroid distance = 3.4808 (7) Å].

## Related literature

For the synthesis, structure and properties of Schiff base complexes, see: Lee *et al.* (2005 ▶); Sriram *et al.* (2006 ▶); Hao (2009 ▶); Bedia *et al.* (2006 ▶). For related structures, see: Tüfekçi *et al.* (2009 ▶); Yazıcı *et al.* (2010 ▶).
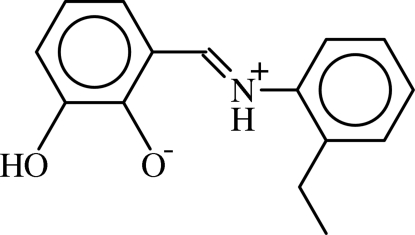


## Experimental

### 

#### Crystal data

C_15_H_15_NO_2_
*M* *_r_* = 241.28
Monoclinic, 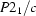

*a* = 7.7482 (4) Å
*b* = 10.8713 (7) Å
*c* = 15.4742 (7) Å
β = 117.380 (3)°
*V* = 1157.42 (11) Å^3^
*Z* = 4
Mo *K*α radiation
μ = 0.09 mm^−1^
*T* = 150 K
0.77 × 0.63 × 0.39 mm

#### Data collection

Stoe IPDS II diffractometer
Absorption correction: integration (*X-RED32*; Stoe & Cie, 2002 ▶) *T*_min_ = 0.945, *T*_max_ = 0.967
10088 measured reflections
2655 independent reflections
2384 reflections with *I* > 2σ(*I*)
*R*_int_ = 0.047

#### Refinement

*R*[*F* ^2^ > 2σ(*F* ^2^)] = 0.042
*wR*(*F* ^2^) = 0.117
*S* = 1.05
2655 reflections
167 parameters
H atoms treated by a mixture of independent and constrained refinement
Δρ_max_ = 0.39 e Å^−3^
Δρ_min_ = −0.45 e Å^−3^

### 

Data collection: *X-AREA* (Stoe & Cie, 2002 ▶); cell refinement: *X-AREA*; data reduction: *X-RED32* (Stoe & Cie, 2002 ▶); program(s) used to solve structure: *SHELXS97* (Sheldrick, 2008 ▶); program(s) used to refine structure: *SHELXL97* (Sheldrick, 2008 ▶); molecular graphics: *ORTEP-3 for Windows* (Farrugia, 1997 ▶); software used to prepare material for publication: *WinGX* (Farrugia, 1999 ▶).

## Supplementary Material

Crystal structure: contains datablocks I, global. DOI: 10.1107/S1600536810002503/ci5022sup1.cif

Structure factors: contains datablocks I. DOI: 10.1107/S1600536810002503/ci5022Isup2.hkl

Additional supplementary materials:  crystallographic information; 3D view; checkCIF report

## Figures and Tables

**Table 1:** Hydrogen-bond geometry (Å, °)

*D*—H⋯*A*	*D*—H	H⋯*A*	*D*⋯*A*	*D*—H⋯*A*
N1—H1⋯O1	0.92 (2)	1.77 (2)	2.5793 (16)	145 (2)
O2—H2⋯O1^i^	0.82	2.13	2.6993 (12)	127

Symmetry code: (i) 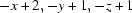.

**Fig. 1.**
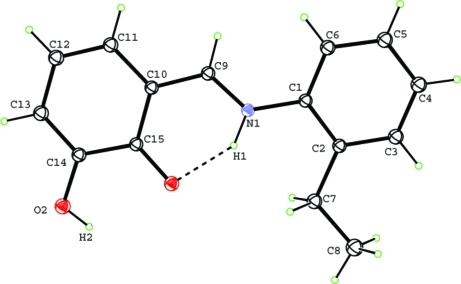
The molecular structure of the title compound, with the atomic numbering scheme. Displacement ellipsoids are drawn at the 30% probability level.

**Fig. 2.**
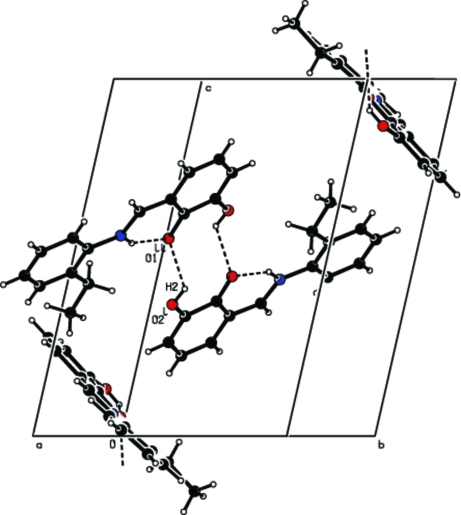
A partial packing diagram of the title compound. Hydrogen bonds are shown as dashed lines

## supplementary crystallographic information

### Comment

Schiff bases are one of the most prevalent and important mixed-donor ligand in coordination chemistry (Lee *et al.*, 2005). Recently, the synthesis, structure and properties of Schiff base complexes have stimulated much more interest for their noteworthy contributions in pharmaceutical and medicinal activities (Sriram *et al.*, 2006; Hao, 2009; Bedia *et al.*, 2006).

The molecule of the title compound exists in a zwitterionic form, with a strong intramolecular N1—H1···O1 hydrogen bond (Fig. 1). The molecule adopts an E configuration with respect to the amine C═N bond with a C10—C9—N1—C1 torsion angle of 176.29 (10)°. The dihedral angle between the two benzene rings is 5.59 (6)°. The C15—O1 [1.2885 (14) Å], C9—N1 [1.3122 (15) Å] and C9—C10 [1.4071 (16) Å] bond lengths are consistent with corresponding values reported for related zwitterionic compounds (Tüfekçi *et al.*, 2009; Yazıcı *et al.*, 2010).

The crystal packing is stabilized by intermolecular O—H···O hydrogen bonds (Table 1) which link the molecules to form dimers. In addition, π–π interactions are observed between C1–C6 (at x,y,z) and C10–C15 (at 1-x,1-y,1-z) benzene rings [centroid-to-centroid distance = 3.4808 (7) Å].

### Experimental

A mixture of 2,3-dihydroxybenzaldehyde (0.5 g, 3.6 mmol) in ethanol (20 ml) and 2-ethylaniline (0.43 g, 3.6 mmol) in ethanol (20 ml) was stirred for 1 h under reflux. Single crystals suitable for X-ray analysis were obtained from ethanol by slow evaporation (yield 85%, m.p. 406-407 K).

### Refinement

Atom H1 was located in a difference map and refined freely. The remaining H atoms were placed in calculated positions and constrained to ride on their parents atoms, with C–H = 0.93-0.97 Å , O–H = 0.82 Å, N–H = 0.92 Å and U_iso_(H) = 1.2U_eq_(C) and 1.5U_eq_(O,C_methyl_).

### Crystal data

**Table:**

C_15_H_15_NO_2_	*F*(000) = 512
*M**_r_* = 241.28	*D*_x_ = 1.385 Mg m^−^^3^
Monoclinic, *P*2_1_/*c*	Mo *K*α radiation, λ = 0.71073 Å
Hall symbol: -P 2ybc	Cell parameters from 14793 reflections
*a* = 7.7482 (4) Å	θ = 2.4–28.0°
*b* = 10.8713 (7) Å	µ = 0.09 mm^−^^1^
*c* = 15.4742 (7) Å	*T* = 150 K
β = 117.380 (3)°	Prism, red
*V* = 1157.42 (11) Å^3^	0.77 × 0.63 × 0.39 mm
*Z* = 4	

### Data collection

**Table:**

Stoe IPDS II diffractometer	2655 independent reflections
Radiation source: fine-focus sealed tube	2384 reflections with *I* > 2σ(*I*)
graphite	*R*_int_ = 0.047
Detector resolution: 6.67 pixels mm^-1^	θ_max_ = 27.5°, θ_min_ = 2.4°
ω scan	*h* = −10→10
Absorption correction: integration (*X-RED32*; Stoe & Cie, 2002)	*k* = −14→14
*T*_min_ = 0.945, *T*_max_ = 0.967	*l* = −20→20
10088 measured reflections	

### Refinement

**Table:**

Refinement on *F*^2^	Primary atom site location: structure-invariant direct methods
Least-squares matrix: full	Secondary atom site location: difference Fourier map
*R*[*F*^2^ > 2σ(*F*^2^)] = 0.042	Hydrogen site location: inferred from neighbouring sites
*wR*(*F*^2^) = 0.117	H atoms treated by a mixture of independent and constrained refinement
*S* = 1.05	*w* = 1/[σ^2^(*F*_o_^2^) + (0.0624*P*)^2^ + 0.418*P*] where *P* = (*F*_o_^2^ + 2*F*_c_^2^)/3
2655 reflections	(Δ/σ)_max_ = 0.001
167 parameters	Δρ_max_ = 0.39 e Å^−^^3^
0 restraints	Δρ_min_ = −0.45 e Å^−^^3^

### Special details

**Table:**

Experimental. 196 frames, detector distance = 80 mm The beam size = 0.8 mm.
Geometry. All esds (except the esd in the dihedral angle between two l.s. planes) are estimated using the full covariance matrix. The cell esds are taken into account individually in the estimation of esds in distances, angles and torsion angles; correlations between esds in cell parameters are only used when they are defined by crystal symmetry. An approximate (isotropic) treatment of cell esds is used for estimating esds involving l.s. planes.
Refinement. Refinement of *F*^2^ against ALL reflections. The weighted *R*-factor wR and goodness of fit *S* are based on *F*^2^, conventional *R*-factors *R* are based on *F*, with *F* set to zero for negative *F*^2^. The threshold expression of *F*^2^ > σ(*F*^2^) is used only for calculating *R*-factors(gt) etc. and is not relevant to the choice of reflections for refinement. *R*-factors based on *F*^2^ are statistically about twice as large as those based on *F*, and *R*- factors based on ALL data will be even larger.

### Fractional atomic coordinates and isotropic or equivalent isotropic displacement parameters (Å^2^)

**Table:**

	*x*	*y*	*z*	*U*_iso_*/*U*_eq_	
C1	0.36021 (16)	0.72765 (10)	0.47504 (8)	0.0173 (2)	
C2	0.48396 (17)	0.81367 (10)	0.54271 (8)	0.0183 (2)	
C3	0.40045 (18)	0.89865 (11)	0.57970 (9)	0.0220 (3)	
H3	0.4791	0.9567	0.6248	0.026*	
C4	0.20240 (18)	0.89846 (11)	0.55063 (9)	0.0240 (3)	
H4	0.1497	0.9558	0.5764	0.029*	
C5	0.08307 (17)	0.81329 (12)	0.48342 (9)	0.0232 (3)	
H5	−0.0498	0.8137	0.4639	0.028*	
C6	0.16097 (17)	0.72722 (11)	0.44512 (9)	0.0208 (3)	
H6	0.0810	0.6698	0.3999	0.025*	
C7	0.69934 (17)	0.81261 (11)	0.57319 (9)	0.0221 (3)	
H7A	0.7175	0.8322	0.5168	0.026*	
H7B	0.7475	0.7297	0.5933	0.026*	
C8	0.82267 (19)	0.90037 (13)	0.65481 (10)	0.0300 (3)	
H8A	0.9564	0.8927	0.6687	0.045*	
H8B	0.8098	0.8806	0.7120	0.045*	
H8C	0.7798	0.9833	0.6353	0.045*	
C9	0.35682 (16)	0.55612 (10)	0.37159 (8)	0.0182 (2)	
H9	0.2228	0.5478	0.3460	0.022*	
C10	0.45648 (16)	0.47823 (10)	0.33700 (8)	0.0179 (2)	
C11	0.34923 (17)	0.39280 (11)	0.26133 (9)	0.0206 (3)	
H11	0.2151	0.3872	0.2367	0.025*	
C12	0.44263 (18)	0.31928 (11)	0.22495 (9)	0.0231 (3)	
H12	0.3725	0.2634	0.1757	0.028*	
C13	0.64667 (18)	0.32805 (11)	0.26231 (9)	0.0225 (3)	
H13	0.7088	0.2782	0.2363	0.027*	
C14	0.75465 (17)	0.40778 (11)	0.33558 (8)	0.0195 (2)	
C15	0.66399 (16)	0.48648 (10)	0.37727 (8)	0.0176 (2)	
N1	0.44559 (14)	0.64003 (9)	0.43849 (7)	0.0171 (2)	
O1	0.76679 (12)	0.56081 (8)	0.44695 (6)	0.0214 (2)	
O2	0.95018 (12)	0.41463 (8)	0.36831 (7)	0.0246 (2)	
H2	0.9964	0.4658	0.4120	0.037*	
H1	0.578 (3)	0.6363 (18)	0.4597 (13)	0.042 (5)*	

### Atomic displacement parameters (Å^2^)

**Table:**

	*U*^11^	*U*^22^	*U*^33^	*U*^12^	*U*^13^	*U*^23^
C1	0.0190 (5)	0.0158 (5)	0.0176 (5)	0.0033 (4)	0.0090 (4)	0.0031 (4)
C2	0.0184 (5)	0.0182 (5)	0.0183 (5)	0.0016 (4)	0.0086 (4)	0.0026 (4)
C3	0.0237 (6)	0.0198 (6)	0.0221 (6)	0.0015 (4)	0.0102 (5)	−0.0015 (4)
C4	0.0258 (6)	0.0234 (6)	0.0252 (6)	0.0080 (5)	0.0138 (5)	0.0012 (5)
C5	0.0173 (5)	0.0264 (6)	0.0263 (6)	0.0049 (4)	0.0103 (5)	0.0032 (5)
C6	0.0189 (6)	0.0209 (6)	0.0211 (6)	0.0008 (4)	0.0077 (4)	0.0008 (4)
C7	0.0184 (5)	0.0241 (6)	0.0241 (6)	−0.0003 (4)	0.0102 (5)	−0.0038 (5)
C8	0.0218 (6)	0.0349 (7)	0.0311 (7)	−0.0053 (5)	0.0103 (5)	−0.0099 (6)
C9	0.0166 (5)	0.0177 (5)	0.0178 (5)	0.0004 (4)	0.0058 (4)	0.0028 (4)
C10	0.0193 (5)	0.0158 (5)	0.0165 (5)	0.0008 (4)	0.0066 (4)	0.0016 (4)
C11	0.0190 (5)	0.0193 (5)	0.0192 (6)	−0.0008 (4)	0.0051 (4)	0.0007 (4)
C12	0.0264 (6)	0.0192 (5)	0.0184 (6)	−0.0012 (4)	0.0058 (5)	−0.0031 (4)
C13	0.0271 (6)	0.0198 (5)	0.0201 (6)	0.0045 (4)	0.0105 (5)	−0.0013 (4)
C14	0.0195 (5)	0.0192 (5)	0.0186 (5)	0.0031 (4)	0.0078 (4)	0.0026 (4)
C15	0.0195 (5)	0.0149 (5)	0.0171 (5)	0.0010 (4)	0.0072 (4)	0.0015 (4)
N1	0.0161 (5)	0.0168 (4)	0.0177 (5)	0.0017 (3)	0.0072 (4)	0.0009 (4)
O1	0.0179 (4)	0.0207 (4)	0.0221 (4)	−0.0007 (3)	0.0063 (3)	−0.0054 (3)
O2	0.0190 (4)	0.0297 (5)	0.0238 (4)	0.0026 (3)	0.0087 (3)	−0.0052 (4)

### Geometric parameters (Å, °)

**Table:**

C1—C6	1.3929 (16)	C8—H8C	0.96
C1—C2	1.4025 (16)	C9—N1	1.3122 (15)
C1—N1	1.4174 (14)	C9—C10	1.4071 (16)
C2—C3	1.3930 (16)	C9—H9	0.93
C2—C7	1.5112 (16)	C10—C11	1.4245 (16)
C3—C4	1.3864 (17)	C10—C15	1.4352 (15)
C3—H3	0.93	C11—C12	1.3617 (17)
C4—C5	1.3818 (18)	C11—H11	0.93
C4—H4	0.93	C12—C13	1.4149 (17)
C5—C6	1.3857 (17)	C12—H12	0.93
C5—H5	0.93	C13—C14	1.3654 (17)
C6—H6	0.93	C13—H13	0.93
C7—C8	1.5194 (17)	C14—O2	1.3607 (14)
C7—H7A	0.97	C14—C15	1.4346 (16)
C7—H7B	0.97	C15—O1	1.2885 (14)
C8—H8A	0.96	N1—H1	0.924 (18)
C8—H8B	0.96	O2—H2	0.82
			
C6—C1—C2	121.54 (10)	H8A—C8—H8C	109.5
C6—C1—N1	120.96 (10)	H8B—C8—H8C	109.5
C2—C1—N1	117.50 (10)	N1—C9—C10	122.56 (10)
C3—C2—C1	117.50 (11)	N1—C9—H9	118.7
C3—C2—C7	122.13 (11)	C10—C9—H9	118.7
C1—C2—C7	120.37 (10)	C9—C10—C11	119.36 (10)
C4—C3—C2	121.31 (11)	C9—C10—C15	119.90 (10)
C4—C3—H3	119.3	C11—C10—C15	120.74 (10)
C2—C3—H3	119.3	C12—C11—C10	120.16 (11)
C5—C4—C3	120.19 (11)	C12—C11—H11	119.9
C5—C4—H4	119.9	C10—C11—H11	119.9
C3—C4—H4	119.9	C11—C12—C13	119.87 (11)
C4—C5—C6	120.14 (11)	C11—C12—H12	120.1
C4—C5—H5	119.9	C13—C12—H12	120.1
C6—C5—H5	119.9	C14—C13—C12	121.66 (11)
C5—C6—C1	119.32 (11)	C14—C13—H13	119.2
C5—C6—H6	120.3	C12—C13—H13	119.2
C1—C6—H6	120.3	O2—C14—C13	119.73 (11)
C2—C7—C8	115.90 (10)	O2—C14—C15	119.44 (10)
C2—C7—H7A	108.3	C13—C14—C15	120.81 (11)
C8—C7—H7A	108.3	O1—C15—C14	120.55 (10)
C2—C7—H7B	108.3	O1—C15—C10	122.71 (10)
C8—C7—H7B	108.3	C14—C15—C10	116.74 (10)
H7A—C7—H7B	107.4	C9—N1—C1	127.62 (10)
C7—C8—H8A	109.5	C9—N1—H1	110.1 (12)
C7—C8—H8B	109.5	C1—N1—H1	122.3 (12)
H8A—C8—H8B	109.5	C14—O2—H2	109.5
C7—C8—H8C	109.5		
			
C6—C1—C2—C3	0.44 (17)	C15—C10—C11—C12	−1.24 (17)
N1—C1—C2—C3	−179.08 (10)	C10—C11—C12—C13	−0.15 (18)
C6—C1—C2—C7	−179.48 (11)	C11—C12—C13—C14	0.92 (19)
N1—C1—C2—C7	0.99 (16)	C12—C13—C14—O2	−178.98 (11)
C1—C2—C3—C4	−0.13 (18)	C12—C13—C14—C15	−0.28 (18)
C7—C2—C3—C4	179.79 (11)	O2—C14—C15—O1	−1.89 (17)
C2—C3—C4—C5	−0.24 (19)	C13—C14—C15—O1	179.41 (11)
C3—C4—C5—C6	0.32 (19)	O2—C14—C15—C10	177.64 (10)
C4—C5—C6—C1	−0.02 (18)	C13—C14—C15—C10	−1.06 (16)
C2—C1—C6—C5	−0.37 (17)	C9—C10—C15—O1	2.05 (17)
N1—C1—C6—C5	179.14 (10)	C11—C10—C15—O1	−178.67 (10)
C3—C2—C7—C8	6.54 (17)	C9—C10—C15—C14	−177.46 (10)
C1—C2—C7—C8	−173.55 (11)	C11—C10—C15—C14	1.81 (16)
N1—C9—C10—C11	−177.37 (10)	C10—C9—N1—C1	176.29 (10)
N1—C9—C10—C15	1.91 (17)	C6—C1—N1—C9	4.29 (18)
C9—C10—C11—C12	178.03 (11)	C2—C1—N1—C9	−176.19 (11)

### Hydrogen-bond geometry (Å, °)

**Table:**

*D*—H···*A*	*D*—H	H···*A*	*D*···*A*	*D*—H···*A*
N1—H1···O1	0.92 (2)	1.77 (2)	2.5793 (16)	145 (2)
O2—H2···O1^i^	0.82	2.13	2.6993 (12)	127

Symmetry codes: (i) −*x*+2, −*y*+1, −*z*+1.
